# A quantitative method for microstructural analysis of myelinated axons in the injured rodent brain

**DOI:** 10.1038/s41598-017-16797-1

**Published:** 2017-11-28

**Authors:** Erik van Tilborg, Caren M. van Kammen, Caroline G. M. de Theije, Maurits P. A. van Meer, Rick M. Dijkhuizen, Cora H. Nijboer

**Affiliations:** 1 Laboratory of Neuroimmunology and Developmental Origins of Disease, University Medical Center Utrecht, Utrecht University, Utrecht, The Netherlands; 2 Biomedical MR Imaging and Spectroscopy Group, Center for Image Sciences, University Medical Center Utrecht, Utrecht University, Utrecht, The Netherlands; 3 Present Address: Department of Medical Microbiology & Infectious Diseases, Erasmus MC, Rotterdam, The Netherlands

## Abstract

MRI studies (e.g. using diffusion tensor imaging) revealed that injury to white matter tracts, as observed in for instance perinatal white matter injury and multiple sclerosis, leads to compromised microstructure of myelinated axonal tracts. Alterations in white matter microstructure are also present in a wide range of neurological disorders including autism-spectrum disorders, schizophrenia and ADHD. Whereas currently myelin quantity measures are often used in translational animal models of white matter disease, it can be an important valuable addition to study the microstructural organization of myelination patterns in greater detail. Here, we describe methods to extensively study the microstructure of cortical myelination by immunostaining for myelin. To validate these methods, we carefully analyzed the organization of myelinated axons running from the external capsule towards the outer layers of the cortex in three rodent models of neonatal brain injury and in an adult stroke model, that have all been associated with myelination impairments. This unique, relatively easy and sensitive methodology can be applied to study subtle differences in myelination patterns in animal models in which aberrations in myelination integrity are suspected. Importantly, the described methods can be applied to determine efficacy of novel experimental treatments on microstructural organization of cortical myelination.

## Introduction

Around 40% of the human brain consists of white matter, which mainly contains myelinated axons that allow rapid transmission of action potentials to distant brain areas^1,2^. Oligodendrocytes are specialized cells in the central nervous system responsible for the myelination of neuronal axons. Proper myelination not only allows efficient axonal signal transduction, but also provides protection and nutritional support for neurons^3,4^. Injury to the myelinated white matter has devastating consequences for neural connectivity, causing (severe) impaired neurological performance and reduced quality of life, as observed in for instance multiple sclerosis (MS), stroke and neonatal white matter injury (WMI)^5–7^. Diffusion tensor imaging (DTI) studies revealed that in various white matter diseases the white matter *microstructure* is affected as indicated by reduced fractional anisotropy (FA) measurements^8–12^. Moreover, recent studies show that similar changes in white matter microstructure are associated with a wide array of psychological disorders, including autism spectrum disorders^13,14^, schizophrenia^15,16^, bipolar disorder^17^ and ADHD^13,18^. As a result, an increased interest has emerged to study myelination in animal models of neurological disease, with the aim of elucidating the mechanisms underlying impaired white matter integrity and to develop novel treatment options to restore myelination under pathological conditions.

Here, we demonstrate using four different rodent models of both neonatal brain injury and adult stroke that staining brain sections for myelin-basic-protein (MBP) enables highly detailed analysis of the microstructural integrity of myelinated axons running from the external capsule in the deep white matter towards the outer cortical layers. In a rat model of neonatal diffuse WMI, a mouse model of neonatal WMI, and in a mouse model of neonatal asphyxia (comprising both neuronal injury and WMI), we show reduced cortical myelination, reduced immunodensity of MBP staining, reduced MBP-positive (MBP+) area and increased coherency of myelinated axons by conventional methods. Moreover, we introduce and validate a novel sensitive method for the detailed quantification of microstructural complexity (i.e. fiber length, number of intersections) of cortical myelination in these models. The method was also reliably applied in an adult rodent model of stroke. In addition to immunofluorescent stainings for MBP, the method can also be applied using DAB stainings and/or other myelin markers, like PLP.

## Materials and Methods

### Animals

All procedures were carried out in accordance with Dutch and European regulations and were approved by the Animal Ethics Committee of Utrecht University. All efforts were made to minimize animal suffering. Animals of both sexes were used in all neonatal models and experimental conditions were divided among all different litters. No sex differences were observed in this study. For the adult MCAO model of stroke, male rats were used (see below).

### Rat model of neonatal diffuse WMI

We used a rat model of neonatal diffuse WMI that was recently introduced^19^. Timed-pregnant Wistar rats (Envigo, Horst, The Netherlands) received intraperitoneal (i.p.) injections of 100 μg/kg LPS (from E. Coli O55:B5; L2880, Sigma-Aldrich, St. Louis, MO) in saline or vehicle on embryonic days (E)18 and E19 of pregnancy. At postnatal day 4 (P4), offspring was placed in a temperature-controlled hypoxic chamber containing 8% O_2_ or in a normoxic environment away from the mother for 140 minutes. Afterwards, animals were returned to their dams. Rats that received fetal saline and postnatal normoxia (referred to as ‘control’, n = 8), rats that received fetal saline and postnatal hypoxia (‘hypoxia only’; n = 7), rats that received fetal inflammation and postnatal normoxia (‘LPS only’; n = 7), and animals that received both fetal inflammation and postnatal hypoxia (‘FIPH’, n = 8) were euthanized at P18 (i.e. 2 weeks post-hypoxia).

### Mouse model of neonatal WMI

Neonatal unilateral WMI was induced in C57BL/6 mice (Envigo) by inducing a hypoxic-ischemic insult, directly followed by an inflammatory stimulus, as adapted from Shen *et al*.^20,21^. Briefly, at P5 ‘HI + LPS’ mouse pups (n = 6) underwent permanent occlusion of the right common carotid artery under isoflurane anesthesia, followed by exposure to 35 minutes of hypoxia (6% oxygen). Immediately after hypoxia, pups received 1 mg/kg LPS i.p. (List Biological Laboratories, Campbell, CA). Sham ‘control’ animals (n = 6) underwent surgical incision only, without occlusion of the carotid artery and without hypoxia. Procedures were carried out under temperature-controlled conditions and afterwards animals returned to their dams. Mice were euthanized at P26 (i.e. 3 weeks post-HI + LPS).

### Mouse model of neonatal asphyxia

Unilateral neonatal hypoxic-ischemic brain injury was induced in C57BL/6 mouse pups (Envigo,) as described earlier^22^. Briefly, at P9 ‘HI’ mouse pups (n = 8) underwent permanent occlusion of the right common carotid artery under isoflurane anesthesia, followed by 45 minutes of hypoxia (10% oxygen). Sham ‘control’ animals (n = 8) underwent surgical incision only, without occlusion of the carotid artery and without hypoxia. All procedures were carried out under temperature-controlled conditions and afterwards animals returned to their dams. Mice were euthanized at P37 (i.e. 4 weeks post-HI). Animals with severe cystic lesions in the cortex (<10%) were not included in the analysis.

### Rat model of adult stroke

Adult male Sprague Dawley rats (280–320 g) underwent unilateral middle cerebral artery occlusion (MCAO) (n = 3) as described earlier^23,24^. Briefly, while anesthetized with isoflurane the right middle cerebral artery was occluded by an intraluminal filament for 90 minutes. Sham-operated ‘control’ animals (n = 3) received a surgical incision under isoflurane anesthesia, without MCAO. Animals were euthanized at 70 days post-MCAO.

### Histology

All animals were sacrificed by an overdose of 250 mg/kg pentobarbital, followed by transcardial perfusion with PBS and 4% PFA in PBS. Brains were post-fixed for 24 hours in 4% PFA and dehydrated by incubation in increasing concentrations of ethanol, before being embedded in paraffin. 8 µm coronal sections were cut at the level of bregma (rats) or at the hippocampal level (corresponding to −1.80 mm from bregma in adult mice (Paxinos brain atlas)).

### Immunofluorescent stainings

Sections were deparaffinized in xylene, followed by rehydration in decreasing ethanol concentrations. For antigen retrieval, sections were heated to 95 °C in 10 mM sodium citrate buffer (pH = 6.0) for 20 minutes. After cooling down and 3 washes in PBS, sections were blocked with 0.1% saponin (Sigma-Aldrich) and 2% BSA (Sigma-Aldrich) for 30 minutes at room temperature, followed by overnight incubation with primary antibodies against MBP (SMI-94; Sternberger Monoclonals Incorporated, Lutherville, MD; mouse; 1:1000), NF200 (N4142; Sigma-Aldrich; rabbit; 1:400) or PLP (ab105784, Abcam, Cambridge, UK; rabbit; 1:400) diluted in PBS. The next day, sections were washed 3 times in PBS and incubated with alexa-fluor conjugated secondary antibodies goat-anti-mouse AF488 and goat-anti-rabbit AF594 (both Life Technologies, Carlsbad, CA, 1:200) for 2 hours at 37 °C. Sections were counterstained with DAPI (1:5000) and embedded using Fluorsave.

Fluorescent stainings were visualized using a Cell Observer microscope, equipped with an AxioCam MRm camera, and using an Endow GFP filter (Filter set 38) (all: Zeiss, Oberkochen, Germany) in a blinded fashion. For measuring thickness of myelinated cortex, one picture of each hemisphere in the rat model was acquired at a 2.5X magnification. In the mouse models, one picture of the hemisphere ipsilateral to the injury was taken at a 2.5X magnification. For all other analyses, in the rat FIPH model one picture of each brain hemisphere was taken using a 20X objective at a fixed distance from the external capsule towards the cortical plate; at the level of the most dorsal part of the striatum adjacent to the external capsule. Both pictures were analyzed and values were averaged. In the mouse P9 HI and P5 HI + LPS models, 3 adjacent 40X pictures were taken at a fixed distance from the external capsule into the cortex superimposing the ipsi- and contralateral hippocampal areas. Pictures were analyzed and values were averaged. In the rat MCAO model, 20X pictures were taken in the perilesional area, and in control animals at a fixed distance from the external capsule into the cortex.

### DAB staining

For 3,3′-Diaminobenzidine (DAB) staining, sections were deparaffinized in xylene, followed by 100% ethanol. Endogenous peroxidase was blocked by incubation in 3% H_2_O_2_ in methanol and sections were then hydrated by decreasing ethanol concentrations. Sections were blocked in 10% normal horse serum in PBS with 0.5% triton X-100, followed by overnight incubation with the primary antibody against MBP (SMI-94, see above; 1:1000). Sections were then washed and incubated in secondary antibody (biotinylated horse-anti-mouse; Vector Laboratories, Peterborough, UK; 1:400), followed by washing in PBS. Biotin was HRP-labeled using a Vectastain ABC kit (Vector Laboratories) according to the manufacturer’s instructions, followed by a washing step in 0.05 M Tris-HCl (pH: 7.6). Staining was performed by incubation in 0.5 mg/ml DAB (Sigma) in 0.05 M Tris-HCl with 0.009% H_2_O_2_. Finally, sections were dehydrated in ethanol and embedded. Stained sections were photographed using an AxioLab.A1 microscope equipped with an AxioCam ICc 5 camera (Zeiss) at 2.5X and 40X magnification at similar brain regions as fluorescent stainings.

### Image analyses

In order to quantify different aspects of white matter microstructure, ImageJ was used^25^, combined with several publically available plugins: OrientationJ^26^ and DiameterJ^27^. Image analyses were performed by researchers blinded to experimental conditions.

#### Thickness of myelinated cortex

The extent of cortical myelination was assessed by measuring the area of the cortical tissue above the external capsule on 2.5X images and by measuring the area of myelinated cortex. The proportion of myelinated cortex was calculated by (myelinated cortex/total cortex) × 100%.

#### MBP immunodensity and MBP+ area

MBP immunodensity was assessed by measuring the mean grey value, subtracted by the mean value of the background staining. Levels of the animals with brain injury are normalized to levels in the model-specific control animals, which were put at 100%. MBP+ area was determined by manually setting a threshold to include all MBP+ stained tissue, followed by measuring the proportion of the field that was positive for MBP staining.

#### Coherency of myelinated axons

Coherency of myelinated axons, as an inverse measure of complexity of white matter microstructure, was assessed using the ‘OrientationJ measure’ function of ImageJ plugin OrientationJ^26^ in the selected whole micrograph.

#### Microstructural complexity

Microstructural complexity of cortical myelination (i.e. fiber length, number of intersections) was assessed using a 2-step approach with the ImageJ plugin DiameterJ^27^. The first step is image segmentation, which is necessary for proper tracing of the myelinated axons and can be achieved by removing the background from the image (Process > Subtract background), applying a threshold to include all MBP+ staining and exclude the background. Images were then saved in 8-bit. Noise/small particles were removed by running the macro listed in supplementary file S1 (Process > Batch > Macro: select input and output folders and paste the macro code). Parameters in the batch code depend on aspects such as microscope objective and camera resolution, and should therefore be optimized by the user. As a second step, various parameters regarding the microstructural organization of myelinated axons are measured by the DiameterJ plugin (Plugins > DiameterJ > DiameterJ 1–018: insert correct scaling, no specific radius), with quantification of the parameters (e.g. fiber length, fiber thickness, intersections, etc.) as output. The software will automatically create maps of the MBP+ fiber tracing (e.g. axial thinning).

### Statistics

Statistics were performed with Graphpad Prism 6.02. When comparing two groups, model-specific control animals were compared to FIPH, HI, HI + LPS or MCAO animals by unpaired t-tests. In case of unequal variances, t-tests with Welch’s correction were used. When comparing more than two groups, one-way ANOVA followed by multiple comparisons with Bonferroni correction was performed.

### Data availability

The datasets generated during and/or analyzed during the current study are available from the corresponding author upon reasonable request.

## Results

### Impaired myelination in rodent models of neonatal brain injury

**Figure 1:**
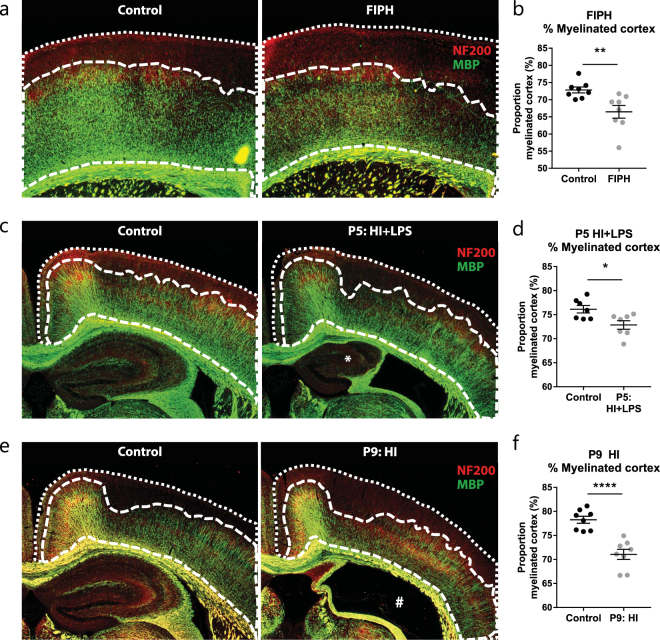
Neonatal brain injury is associated with reduced myelination in outer cortical layers. (**a**) Representative micrographs of a control rat (left panel) and a rat exposed to fetal inflammation (LPS at E18 + E19) and postnatal hypoxia at P4 (FIPH model; right panel) stained for axonal marker NF200 (red) and myelin marker MBP (green). (**b**) Bar graph showing that rats exposed to FIPH display reduced extent of cortical myelination (n = 8 per group). (**c**) Representative images of sham-operated control mice (left) or mice exposed to HI + LPS at P5 (right) stained for MBP (green) and NF200 (red). *Enlarged ventricle and reduced ipsilateral hippocampal volume which is typically observed in this model. (**d**) Bar graph illustrating that HI + LPS mice show reduced extent of cortical myelination (n = 6 per group). (**e**) Representative images of sham-operated control mice (left) or mice exposed to HI on P9 (right) stained for MBP (green) and NF200 (red). #: severely injured hippocampal area characteristic for this model (**f**) Bar graph illustrating that HI mice show reduced extent of cortical myelination (n = 8 per group). Data represent mean ± SEM; *p < 0.05; **p < 0.01; ****p < 0.0001.

In order to study cortical myelination after neonatal brain injury in great detail, brain sections were stained for MBP and various analyses were performed. Myelination was investigated in three rodent models: a rat 2-hit model of neonatal diffuse WMI (fetal inflammation and postnatal hypoxia (FIPH)), a P5 mouse 2-hit model of neonatal unilateral WMI (hypoxia-ischemia (HI) + LPS), and a P9 mouse model of neonatal unilateral asphyxia comprising both grey and white matter injury (HI). In the developing rodent brain, cortical myelination forms a gradient from densely myelinated axons near inner white matter structures such as the external capsule, towards sparsely myelinated outer layers of the cortex. In order to study whether the extent of cortical myelination is affected in rodent models of neonatal brain injury, we measured the proportion of cortical tissue that contains myelinated axons (Fig. 1a,c,e). We observed a reduced percentage of myelinated cortex in all three models of neonatal brain injury (FIPH: p = 0.007; P5 HI + LPS: p = 0.017; P9 HI: p < 0.0001) (Fig. 1).

**Figure 2:**
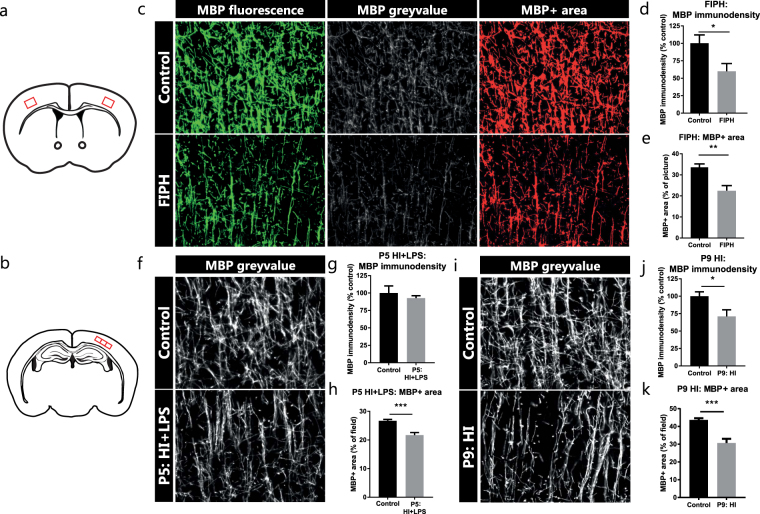
MBP immunodensity and MBP+ area in rodent models of neonatal brain injury. (**a**) Illustration of coronal section of rodent brain with the cortical locations (red squares) of obtained micrographs for the rat FIPH model. (**b**) Illustration of coronal section of rodent brain with cortical locations (red squares) of obtained micrographs for both mouse models of neonatal unilateral brain injury. (**c**) Representative pictures of MBP+ axons in the cortex of control rats (upper panels) and rats exposed to fetal inflammation (LPS at E18 + E19) and postnatal hypoxia (P4) (FIPH; lower panels). Left: original fluorescent signal of MBP staining (green); middle: grey-scale image of MBP fluorescent signal (white) from which immunodensity is measured; right: threshold set to include MBP+ signal (red) and exclude background to measure MBP+ area. (**d**,**e**) Bar graphs showing that rats exposed to FIPH display reduced MBP immunodensity as well as a decrease in MBP+ area (n = 8 per group). (**f**,**i**) MBP fluorescent signal (white) in the cortex of control mice exposed to sham-operation (upper panels) or mice exposed to HI + LPS at P5 (lower panel, **f**) or to HI at P9 (lower panel, **i**). (**g**) No change was observed in MBP immunodensity in mice exposed to HI + LPS at P5 compared to control mice (n = 6 per group). (**h**) P5 HI + LPS animals showed a decrease in MBP+ area compared to sham-operated controls (n = 6 per group). (**j**,**k**) Compared to controls, mice exposed to HI at P9 showed reduced MBP immunodensity (n = 8 per group) as well as a decrease in MBP+ area (n = 8 per group). Data represent mean ± SEM; *p < 0.05; **p < 0.01; ***p < 0.001.

To investigate whether differences exist in the quantity of myelination, we measured the immunodensity of the MBP fluorescent signal in the cortex (Fig. 2a,b). Furthermore, the MBP+ area of each picture was quantified. In the rat model of diffuse WMI, we observed a reduction in both the MBP immunodensity (p = 0.029) and in the MBP+ area (p = 0.002) compared to control animals (Fig. 2c–e). Compared to sham-operated controls, mice exposed to HI + LPS at P5 showed no changes in MBP immunodensity (p = 0.523), but did show a reduction in MBP+ area (p = 0.001) (Fig. 2f–h). Furthermore, the mouse model of neonatal asphyxia at P9 caused reductions in both MBP immunodensity (p = 0.026) and MBP + area (p = 0.001) (Fig. 2i–k).

### Coherency as a measure of complexity in the organization of myelination patterns

**Figure 3:**
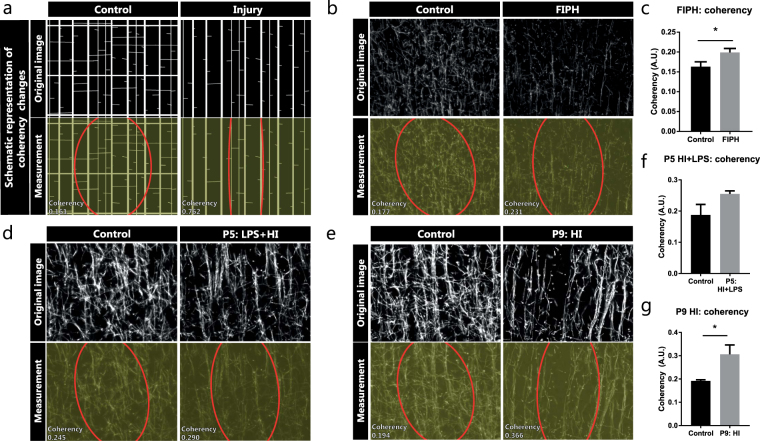
Coherency as an inverse measure of complexity in the organization of myelinated axons. (**a**) Schematic representation of how a less complex organization causes increased coherency between myelinated axons. (**b**,**d**,**e**) MBP fluorescent signal (upper panels) and coherency measurement (lower panels) with coherency ellipse (red) of model-specific control animals (left) and rats exposed to fetal inflammation (LPS at E18 + E19) and postnatal hypoxia (P4) (FIPH; right **b**), or mice exposed to HI + LPS at P5 (right **d**), or mice exposed to HI at P9 (right **e**). (**c**) Bar graph showing increased coherency of myelinated axons in rats exposed to FIPH (n = 8 per group). (**f**) Mice exposed to HI + LPS at P5 do not show statistically significant changes in coherency of myelinated axons in the cortex (n = 6 per group). (**g**) Bar graph illustrating increased coherency of myelinated axons in mice exposed to HI at P9 (n = 8 per group). Data represent mean ± SEM; *p < 0.05.

In order to investigate changes in the organization of cortical myelination in greater detail, we measured the coherency of myelinated axons as an inverse measure of arborization/complexity of myelination patterns (Fig. 3a). Compared to control animals, rats exposed to FIPH showed an increased coherency in axonal myelination, indicative of reduced complexity (p = 0.041) (Fig. 3b,c). These results indicate that axons running from the deep white matter towards the outer cortex (vertically oriented) remained relatively spared, whereas myelination of perpendicular oriented intersecting axons (horizontally oriented) are most severely affected by exposure to FIPH. Furthermore, whereas mice exposed to HI + LPS at P5 did not show a statistically significant increase in coherency of MBP+ axons (p = 0.105) (Fig. 3d,f), mice exposed to HI at P9 did show an increased coherency (p = 0.029) versus their sham-operated controls (Fig. 3e,g).

### Reduced microstructural complexity of myelination patterns after neonatal brain injury

**Figure 4:**
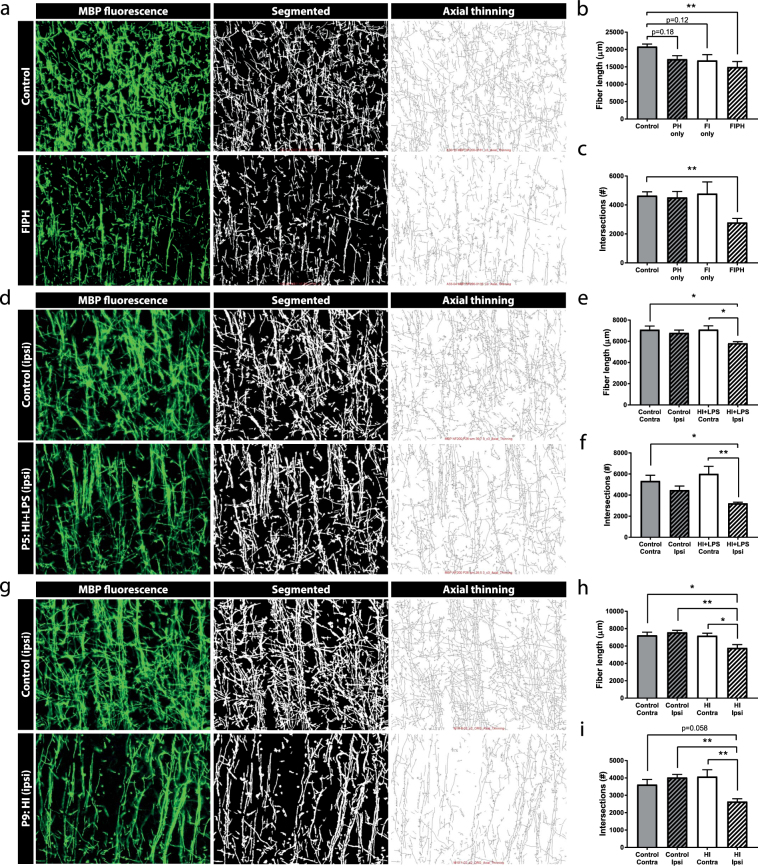
Segmentation of MBP staining allows detailed analysis of microstructural integrity of myelinated cortex. (**a**,**d**,**g**) The original image of MBP fluorescent signal (left), the segmented image extracted from the original image (middle), and the axial thinning map as calculated from the segmented image (right). Upper panels show model-specific control animals and lower panels show (**a**) rats exposed to fetal inflammation (LPS at E18 + E19) and postnatal hypoxia (P4) (FIPH); (**d**) mice exposed to HI + LPS at P5 or (**g**) mice exposed to HI at P9. Different parameters of fiber organization can be calculated from these maps. (**b**,**c**) Single insults (postnatal hypoxia (PH) only, n = 7; fetal inflammation (FI) only, n = 7) do not significantly affect the fiber length or the number of intersections, but the combination of fetal inflammation and postnatal hypoxia (FIPH) (n = 8) causes a reduction in both fiber length and intersections compared to control animals (n = 8). (**e**,**f**) The ipsilateral (injured; ipsi) hemisphere of mice exposed to HI + LPS at P5 shows decreased length and reduced number of intersections of myelinated axons compared to the contralateral (contra) hemisphere of both control and HI + LPS animals (n = 6 per group). (**h**,**i**) The ipsilateral (injured) hemisphere of mice exposed to HI at P9 shows decreased length and reduced number of intersections of myelinated axons compared to the contralateral hemisphere and the hemispheres of the control animals (a trend is observed vs. contralateral hemisphere of control for number of intersections) (n = 8 per group). Data represent mean ± SEM; *p < 0.05; **p < 0.01.

In order to get more detailed measures of the microstructural complexity of myelination patterns in the rodent cortex, myelinated axons were digitally tracked and analyzed. Various parameters regarding the organization of the white matter microstructure were calculated, including fiber length and the number of intersections. Compared to controls, only rats exposed to both FI + PH hits showed a reduction in fiber length (p = 0.008) and a reduction in the number of intersections of MBP+ axons (p = 0.007) (Fig. 4a–c). Single insults (i.e. PH only or FI only) did not affect microstructural organization of myelination: PH only did not affect fiber length (p = 0.182) or the number of intersections (p > 0.999), nor did only FI negatively affect either parameter (p = 0.116; p > 0.999, respectively) (Fig. 4b,c). Similarly, the injured (ipsilateral) hemisphere of mice exposed to HI + LPS at P5 showed reductions in fiber length and the number of intersections compared to the contralateral hemisphere or compared to the ipsilateral hemisphere of control littermates (fiber length: p = 0.020, p = 0.013; intersections: p = 0.022, p = 0.002, respectively) (Fig. 4d–f). Also, mice exposed to HI at P9 displayed statistically significant (or a trends towards) reductions in both fiber length and the number of intersections compared to the contralateral hemisphere (fiber length: p = 0.014; intersections: p = 0.004) or compared to either hemisphere of control animals (contra: p = 0.011, p = 0.058; ipsi: p = 0.002, p = 0.005, respectively) (Fig. 4g–i). Altogether, these results indicate that measures like fiber length and the number of intersections provide a sensitive and specific method for quantifying complexity of myelination in the rodent cortex.

### Reduced microstructural complexity of myelination patterns in a stroke model in adult rats

**Figure 5:**
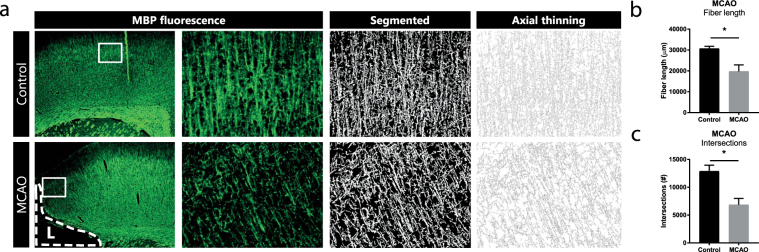
Segmentation of MBP staining allows detailed analysis of the microstructural organization in an adult rat model of stroke by MCAO. (**a**) In brain sections of adult rats, MBP staining allows segmentation and proper tracing of myelinated axons. (**b**,**c**) At 70 days post-MCAO reduced fiber length and reduced number of intersections are observed in the perilesional area of MCAO animals (n = 3), compared to controls (n = 3). L = cortical lesion caused by MCAO. Data represent mean ± SEM; *p < 0.05.

To find out whether this same methodology to quantify detailed aspects of myelin organization can be applied in adult rodent models of brain injury, we quantified MBP+ fiber length and the number of intersections in adult rats at 70 days post-MCAO and compared them with sham-operated control animals. Also in this adult stroke model, segmentation and tracing of myelinated axons in the perilesional area (MCAO animals) or cortical areas (in control animals) was reliably performed (Fig. 5a). We observed that at 70 days post-MCAO, myelination was still impaired on a microstructural level in the perilesional area: both fiber length and the number of intersections were reduced in MCAO rats compared to control animals (p = 0.029; p = 0.018, respectively) (Fig. 5b,c).

### Quantification of microstructural organization of cortical myelination using other stainings

**Figure 6:**
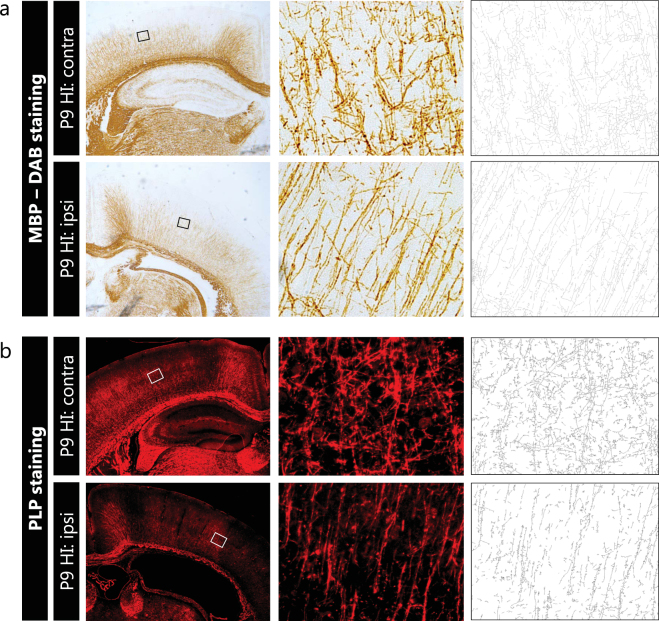
Segmentation of myelinated axons is also applicable on MBP-DAB staining and immunofluorescent PLP staining. (**a**) Contralateral hemisphere (upper panels) and ipsilateral hemisphere (lower panels) of mice exposed to unilateral HI at P9, at 2.5X (left) and 40X (middle) magnification. MBP-DAB staining allowed reliable segmentation and tracing of myelinated axons (right). (**b**) Contralateral hemisphere (upper panels) and ipsilateral hemisphere (lower panels) of mice exposed to unilateral HI at P9, at 2.5X (left) and 40X (middle) magnification. A fluorescent staining for myelin component PLP allowed segmentation and tracing of myelinated axons (right). Squares in 2.5X photograph represent the location of higher magnification images (middle).

To study the applicability of the proposed methodology using other types of stainings in comparison to immunofluorescent stainings or to other myelin markers than MBP, we applied the same analysis protocol on a DAB-staining for MBP (Fig. 6a) and on a fluorescent staining for myelin proteolipid protein (PLP) (Fig. 6b), respectively. Using brain sections of mice exposed to HI at P9, the segmentation and tracing of myelinated axons was successful for both the MBP-DAB staining and the fluorescent PLP staining (Fig. 6). These results highlight the versatility of the proposed methodology and indicate that the analysis procedure can be applied in other (myelin) stainings.

## Discussion

In the present study, we describe a novel methodology to assess various aspects of white matter microstructure in great detail. The described procedures allow sensitive assessment of several parameters regarding the extent and complexity of cortical myelination patterns. The procedures were applied and validated using four different rodent models of neonatal and adult brain injury, all of which have been reported to cause myelination deficits in rats or mice^19–23^. More specifically, in the neonatal models brain sections stained for myelin marker MBP were analyzed to quantify the percentage of myelinated cortex, MBP immunodensity and MBP+ area, as commonly used measures for the extent of myelination. Furthermore, detailed image analysis allowed assessment of the coherency of myelinated axons. Additionally, a novel sensitive methodology was applied to assess fiber length and number of intersections between MBP+ axons as measures of *microstructural organization*. Even in the most subtle model (HI + LPS in P5 mice), in which no significant differences in MBP immunodensity or coherency of myelinated axons were shown, a clear reduction was observed in fiber length and number of intersections, further highlighting the sensitivity of the described methods. In addition, we confirmed the applicability of the methodology in an adult rat model of stroke. Moreover, we show that the described methodology to quantify fiber length and the number of intersections can also be applied to non-fluorescent stainings (e.g. MBP-DAB immunostaining) and to other myelin markers (e.g. PLP).

Currently, immunodensity and area measurements of myelin markers are considered the ‘gold standard’ to quantify myelin deviations in various experimental rodent models^28–34^. Although these measures accurately reflect the amount of myelination, relatively subtle deviations in the microstructural integrity of myelinated axons may be overlooked when only assessing immunodensity and/or area measurements of myelin markers (as illustrated in the P5 HI + LPS mouse model). Here, we show that assessment of microstructural integrity is a highly sensitive method to quantify deviations in cortical myelination and organization, thereby providing valuable additional information to immunodensity and area measurements. Considering the high relevance of white matter microstructure for understanding a wide variety of neurological disorders, it will be crucial to take into account these more detailed analyses of myelination patterns in experimental models of brain disease, making these procedures of high translational value. Fiber length and number of intersections of MBP+ axons were the most robust measures in the evaluated rodent models, but the described methodology can provide information on various additional parameters regarding microstructural organization (e.g. porosity), which might be of value and interest in other brain disease models.

In contrast to stereology and MRI techniques, the here described method allows assessment of myelination in a single coronal (2D) plane. The strength of detailed analysis of myelin staining is that it provides additional insight in the quantity and microstructural organization of myelinated axons. A downside can be that conclusions regarding volumetric changes in white matter anatomy or myelination will be difficult; although our analyses can be performed on sections throughout the rodent brain and in various brain regions, as long as the myelinated axons are not too densely organized such as in the corpus callosum. Stereology, a more suitable method to assess volume changes, has previously been performed on electron microscopy (EM) images throughout the brain to accurately assess myelin sheath thickness^35,36^. However, stereology at a cellular level to assess volumetric changes in the brain would be more challenging, due to the many different brain regions that are functionally, structurally and anatomically distinct. Total gray vs. white matter volume changes can be more straightforwardly determined by whole-brain MRI. Furthermore, MRI-based DTI studies over the past years revealed changes in white matter microstructure, as reflected by altered FA values, in patients suffering from a wide range of neurological disorders, including autism-spectrum disorders, schizophrenia and bipolar disorder^13–18^. MRI can also be applied on rodents. However, the relatively low spatial resolution of MRI makes this technique less appropriate for detection in small (sub)regions as compared to microscopic analysis of MBP immunostaining. In an earlier study performed by our group no FA changes were observed in the FIPH rat model at P30 using MRI, whereas MBP staining did reveal a significant reduction in cortical myelination^19^. In addition, DTI can yield ambiguous results: whereas lowered FA values in white matter are usually interpreted as a reduction in structural integrity, low FA values in *cortical areas* typically reflect a more complex and less coherent microstructural organization. In line, regionally increased FA values as observed in human preterm infants and patients with autism spectrum disorders^37–40^ may be explained by increased coherency of myelinated axons as observed in animal models of brain injury^19^. The more advanced diffusion kurtosis imaging (DKI) has been suggested to be more suitable for measuring (changes in) microstructural integrity in complex, rather than coherent fiber arrangements^41^, although the relationship between diffusion behavior and tissue microarchitecture appears highly multifaceted^42^. Compared to immunostaining, MRI studies are expensive, require a highly specialized infrastructure and provide indirect measures of microstructural organization. Nevertheless, for clinical translation, MRI techniques remain highly valuable.

The here described method is relatively fast and easy-to-apply as it relies on basic immunofluorescent staining techniques, widely used epifluorescence microscopy and open source image analysis software. The described procedures on microstructural analysis can also be used on non-fluorescent (e.g. DAB) stainings, although it should be noted that standard immunodensity measures are not accurate on DAB stainings. Additionally, in the present study we focused on cortical myelination, but the described methodology may also be applied to other brain regions such as the *cerebellar* cortex. Furthermore, the proposed methodology can also be used on other myelin markers (e.g. PLP), or on markers of other types cell types that form networks. It should be taken into account that in order to successfully apply the described methodology in practice, it is crucial that all procedures (e.g. cutting sections) are carried out as standardized as possible for all samples. For instance, the angle of sectioning and the coronal plane of which sections are used may affect the measurements of microstructural integrity of myelinated cortical axons. It should be noted that based on the proposed image analysis procedures, conclusions on *qualitative* aspects of myelination such as myelin sheath length, paranodal length and myelin thickness cannot be drawn. In order to accurately examine such parameters, in particular myelin sheath thickness, EM remains the method of choice. EM allows the measurement of G-ratios, a measure of myelin thickness relative to axon diameter which is the most accurate way of estimating myelin integrity at a molecular level. However, the microstructural organization of cortical myelination is a measure independent of myelin sheath thickness, i.e. there may be a reduction in the number of myelinated axons without affecting the thickness of the existing myelin sheaths, and conversely, the overall myelin sheath thickness may be reduced without affecting the number and organization of myelinated axons. The described methodology is proposed as a sensitive addition to existing methods to quantify myelination at more microscopical level than MRI, but at a more macroscopical level compared to EM, adding more depth to commonly used techniques such as MBP immunodensity or area measurements. To gain a total understanding of myelination in a particular animal model, different techniques should be combined: MRI could be used to estimate total myelinated white matter volumes, histology allows assessment of myelin quantity and myelin organization in specific brain areas, and EM enables assessment of myelin quality at a molecular level.

To conclude, immunostainings for myelin markers followed by different methods of image analysis yield highly detailed information regarding the extent and microstructural integrity of cortical myelination in rodents. The detailed analysis of microstructural integrity of cortical myelination may be used to study myelination in white matter diseases, such as neonatal WMI or multiple sclerosis. Additionally, it may be used in other models of neurological impairments. For example, if a specific gene has been implicated in a neurological disorder, e.g. autism-spectrum disorders, a detailed investigation of myelination in a knockout animal may reveal whether symptoms are mediated by white matter changes. Microstructural investigation of cortical myelination may aid the development of novel treatment options aimed at protecting myelination or promoting remyelination^43,44^. Furthermore, adverse effects of novel treatments for other diseases (e.g. chemotherapy^45^, radiation therapy^46^) on myelination may be sensitively characterized. Overall, the detailed analysis of myelin staining in the rodent brain may significantly contribute to preclinical white matter research.

## Electronic supplementary material

Supplementary Information
