# Supplementary material for: A quantitative method for microstructural analysis of myelinated axons in the injured rodent brain
Source: Sci Rep. 2017 Nov 28;7:16492. doi: 10.1038/s41598-017-16797-1 (PMC5705703; doi:10.1038/s41598-017-16797-1)
Supplement: Supplementary file 1 — Supplementary Information [file 41598_2017_16797_MOESM1_ESM.doc]

**A quantitative method for microstructural analysis of myelinated axons in the injured rodent brain**

Erik van Tilborg, Caren M. van Kammen, Caroline G. M. de Theije, Maurits P.A. van Meer, Rick M. Dijkhuizen, Cora H. Nijboer

**Supplementary file S1**

Macro used to segment 20X pictures of rat cortex stained for MBP

Paste macro in the process>batch>macro function of ImageJ Parameters should be optimized by user

run("Invert");

run("Analyze Particles...", "size=0-400 pixel circularity=0.40-1.00 show=Nothing clear add");

roiManager("Show All without labels"); count=roiManager("count"); array=newArray(count); for(i=0; i<count;i++) {

array[i] = i;

}

roiManager("Select", array);

roiManager("Combine");

setBackgroundColor(255, 255, 255);

run("Clear", "slice");

roiManager("Deselect");

roiManager("Delete");

run("Select All");

run("Invert");

close();

1

Macro used to segment 40X pictures of mouse cortex stained for MBP Paste macro in the process>batch>macro function of ImageJ Parameters should be optimized by user

run("Invert");

run("Analyze Particles...", "size=0-100 pixel circularity=0.00-1.00 show=Nothing clear add");

roiManager("Show All without labels");

run("Invert");

count=roiManager("count");

array=newArray(count);

for(i=0; i<count;i++) {

array[i] = i;

}

roiManager("Select", array);

roiManager("Combine");

setForegroundColor(255, 0, 0);

run("Fill", "slice");

roiManager("Deselect");

roiManager("Delete");

setAutoThreshold("Default dark"); //run("Threshold..."); setThreshold(129, 255); setOption("BlackBackground", false); run("Convert to Mask"); run("Invert");

2
